# Characterization of Ni_3_Sn intermetallic nanoparticles fabricated by thermal plasma process and catalytic properties for methanol decomposition

**DOI:** 10.1080/14686996.2019.1622447

**Published:** 2019-06-17

**Authors:** Ya Xu, Huixin Jin, Toshiyuki Hirano, Yoshitaka Matsushita, Jianxin Zhang

**Affiliations:** a Center for Green Research on Energy and Environmental Materials, National Institute for Materials Science, Ibaraki, Japan; b Key Laboratory for Liquid-Solid Structural Evolution & Processing of Materials (Ministry of Education), School of Materials Science & Engineering, Shandong University, Jinan, P. R. China; c Research Network and Facility Services Division, National Institute for Materials Science, Ibaraki, Japan

**Keywords:** Intermetallic compounds, Ni_3_Sn, nanoparticles, methanol decomposition, thermal plasma, hydrogen production, 50 Energy Materials, 205 Catalyst / Photocatalyst / Photosynthesis, 106 Metallic materials, 503 TEM, STEM, SEM

## Abstract

The intermetallic compound Ni_3_Sn has potential for application in hydrogen production as a catalyst. Herein, we synthesized Ni_3_Sn nanoparticles through a thermal plasma process. We characterized the nanoparticles by synchrotron radiation X-ray diffraction and transmission electron microscopy analyses, and analyzed their catalytic properties for methanol decomposition in a temperature range of 513 to 793 K. The Ni_3_Sn nanoparticles showed a higher selectivity to H_2_ and CO than pure Ni nanoparticles, but a relatively lower catalytic activity for methanol decomposition compared to pure Ni nanoparticles. Density functional theory calculations revealed that the activation energy barrier for CO dissociation on Ni_3_Sn (001) was 396 kJ/mol, which was higher than that for Ni (111). Moreover, the activation energy barrier for OH formation on Ni_3_Sn (001) was 229 kJ/mol, which was significantly higher than that for Ni (111). This supported the experimental results and confirmed that the Ni_3_Sn catalyst suppresses the formation of carbon and H_2_O, compared to Ni catalyst.

## GRAPHICAL ABSTRACT

**Figure:**
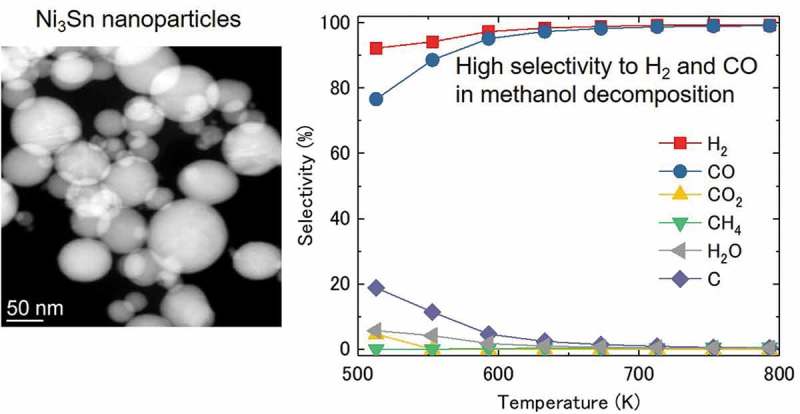

## 1. Introduction

Intermetallic compounds (IMCs) with ordered crystal structures are known to exhibit unique physical and mechanical properties that alloys and pure metals do not possess, such as shape memory effect [1], superconductivity [2], and excellent strength at high temperatures [3,4]. IMCs have potential as catalysts or catalyst precursors owing to their specific crystal and electronic structures [5,6]. The catalytic properties of many IMCs for various chemical reactions have been investigated [7–12]. For example, the catalytic properties of Pt-Ge IMCs for the hydrogenation of 1,3-butadiene were investigated by Komatsu et al. [9]. They found that Pt_3_Ge shows higher selectivity toward butenes, which suggested that the electron transfer from Ge to Pt5d orbitals as well as the difference in the geometrical environment of Pt active sites may contribute to the catalytic performance of Pt-Ge IMCs, based on X-ray photoelectron spectroscopy (XPS) analysis. The catalytic properties of Ni-Sn IMCs for acetylene hydrogenation were investigated by Onda et al. [10]. They reported that Ni_3_Sn shows a higher selectivity for the partial hydrogenation of acetylene, which may be related to the change in the electron density of the Ni valence band at the Fermi level. Tsai et al. [12] found that PdZn exhibits a high selectivity for methanol steam reforming, similar to Cu. They confirmed via energy band calculations and XPS analysis that PdZn has a similar valence electron density of states as pure Cu, and concluded that the catalytic properties of IMCs may be governed by their valence band structures. Among them, Ni-Sn IMCs, such as Ni_3_Sn, Ni_3_Sn_2_, and Ni_3_Sn_4_, are expected to play an important role in improving the catalytic performance of Ni-based catalysts for hydrogen production. For example, it was shown that the addition of Sn to Ni catalysts increased selectivity toward hydrogen without decreasing hydrogen production rate in aqueous hydrocarbon reforming, and the improved catalytic performance was attributed to the formation of Ni_3_Sn in the Ni-Sn catalysts [13,14]. The carbon tolerance of Ni catalysts for steam reforming of hydrocarbon can be improved by synthesizing Ni-Sn surface alloys [15,16]. However, these researches on Ni-Sn catalysts limited to the Ni-enriched composition (Sn < 5 at%), and experimental data on the catalytic properties of Ni_3_Sn itself are scarce in the literature. Recently, we fabricated single-phase Ni_3_Sn and Ni_3_Sn_2_ ingots by using a high-frequency vacuum melting method and examined their catalytic properties for methanol decomposition using the crushed ingot samples (<75 μm in diameter) [17,18]. We found that both Ni_3_Sn and Ni_3_Sn_2_ showed a high selectivity toward hydrogen and carbon monoxide over a wide temperature range; Ni_3_Sn exhibited much higher catalytic activity than Ni_3_Sn_2_. These results suggest that Ni_3_Sn is a suitable catalyst for hydrogen production among the Ni-Sn IMCs. Because the specific surface area of bulk Ni_3_Sn samples is too small to achieve efficient catalytic activity in practical applications, it is, however, necessary to develop Ni_3_Sn nanoparticles with a larger surface area in order to pursue both high catalytic activity and high selectivity for hydrogen production.

**Figure 10.**
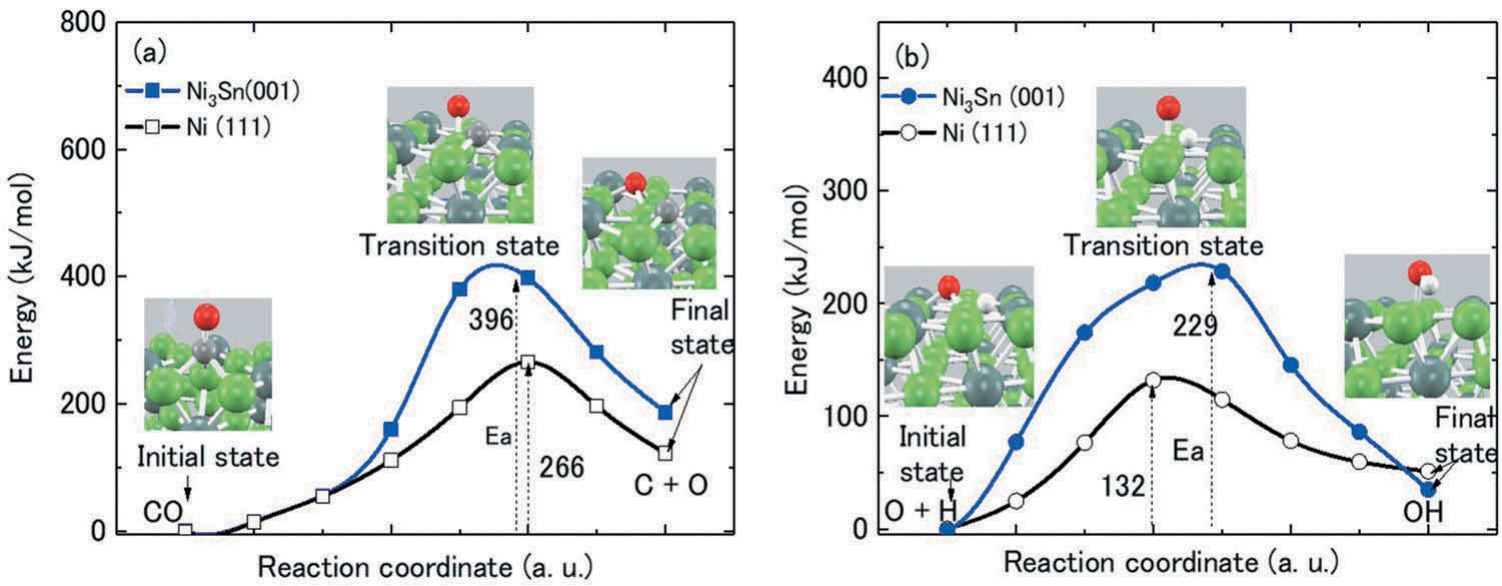
Activation energy barriers for CO dissociation (a) and OH formation (b) on Ni_3_Sn (001) and Ni (111) surfaces. The inserts show the geometries of the initial, transition, and final states. The O atom is in red, C in gray, H in white, Ni in green, and Sn in blue-gray colors.

The thermal plasma process is an effective method for fabricating nanoparticles of alloys and IMCs because the condensation of gasified phases from an extremely high temperature (>10,000 K) in this process is conducive to the formation of homogenous nanoparticle phases with a high degree of crystallinity. We have successfully fabricated Ni-Al and Ni-Fe-Mg nanoparticles using the thermal plasma method in previous studies [19–21]. In this study, we synthesize Ni_3_Sn nanoparticles through the thermal plasma process and examine their catalytic properties for methanol decomposition. A detailed characterization of the fabricated Ni_3_Sn nanoparticles are performed by transmission electron microscopy (TEM) and powder X-ray diffraction using a synchrotron radiation source (SR-XRD). Their catalytic properties are examined by a comparison with those of pure Ni nanoparticles under the same experimental condition. We demonstrate that the Ni_3_Sn nanoparticles show a significantly higher selectivity toward H_2_ and CO in a temperature range of 513 to 793 K and a higher hydrogen production ability at high temperatures compared to pure Ni nanoparticles. These results are analyzed by calculating the activation energy barriers of the related elementary reactions based on the density functional theory (DFT).

## 2. Experimental details

### 2.1 Preparation of Ni_3_Sn and Ni nanoparticles

Ni_3_Sn and Ni nanoparticles were synthesized using a radio-frequency induction thermal plasma setup (Nisshin Engineering Inc., Japan). The raw materials used were Ni fine powder (99.9 wt%, 2–3 μm in diameter) and Sn atomized powder (99.9 wt%, <38 μm in diameter) (Kojundo Chem. Co. Lab., Japan). The actual composition of the synthesized Ni_3_Sn nanoparticles was examined to be Ni-21.3 at% Sn by fluorescence X-ray analysis (Rigaku Co. Ltd., ZSX Primus Ⅱ), showing that the Sn concentration was slightly lower than the stoichiometric composition, 25 at% in Ni_3_Sn.

### 2.2 Methanol decomposition reaction tests

The catalytic reaction tests were carried out in a conventional fixed-bed flow reactor using a quartz tube with an inner diameter of 8 mm as described in our previous works [22,23]. The amount of catalyst used was 50 mg. Prior to the reaction, the catalyst was reduced at 773 K for 1 h in a flow of hydrogen (30 mL/min) and nitrogen (5 mL/min) (all gas volumes are presented as values at standard temperature and pressure (273 K and 0.1 MPa)). After the reduction of the catalyst was complete, a flow of nitrogen (30 mL/min) was introduced to flush the hydrogen, and the temperature was reduced to 703 K. As the next step, methanol was introduced by a micro-pump at a liquid flow rate of 50 μL/min (liquid hourly space velocity (LHSV) of 30 h^−1^) together with a nitrogen flow of 30 mL/min. The catalytic reaction tests were performed in a temperature range of 513–793 K by stepwise heating at intervals of 40 K. The composition of the outlet products was analyzed using two on-line gas chromatographs (GC) with thermal conductivity detectors (TCD) (GL Science, Japan, GC323) after equilibrating for 30 min at each temperature. The total flow rate of the outlet gases was measured using a flow meter (Mesa Laboratories, Defender530) during each GC measurement.

The production rate of each gaseous product was evaluated in terms of the Brunauer–Emmett–Teller (BET) specific surface areas of the nanoparticles after pre-reduction at 773 K for 1 h by the following equation:

(1) $$R_{i}=(C_{i}\;\times \;F_{t})/A_{BET}$$

where R_i_ is the production rate of product i in mol min^−1^ m^−2^-cat; C_i_ is the volume fraction of product *i* in the total outlet gases excluding CH_3_OH and H_2_O; and F_t_ is the total flow rate of outlet gases excluding CH_3_OH and H_2_O in mol min^−1^ g^−1^-cat. A_BET_ is the BET specific surface area of the nanoparticles after pre-reduction at 773 K for 1 h (m^2^ g^−1^). The selectivities toward H_2_ (S_H_2__), H_2_O (S_H_2__O__), and the other carbon-contained products (CO, CO_2_, CH_4_, and C) (S_x_) were calculated by the following equations.

(2) $$S_{H_{2}}=R_{H_{2}}/(R_{H_{2}}\;+\;{R_{CH}}_{{\;}_{4}}\;+\;R_{H_{2}O})\times 100$$

(3) $$S_{H_{2}O}=R_{H_{2}O}/(R_{H_{2}}\;+\;{R_{CH}}_{{\;}_{4}}\;+\;R_{H_{2}O})\times 100$$

(4) $$S_{x}=R_{x}/(R_{CH_{4}}\;+\;R_{C}\;+\;R_{CO}\;+\;R_{CO_{2}})\times 100$$

where R_i_ represents the production rate of each respective molecule.

### 2.3 Catalyst characterization

The BET specific surface areas of the nanoparticles were measured by nitrogen adsorption using a surface area analyzer (Micrometritics,  Instrument Corp., USA, ASAP 2020). The surface morphologies were analyzed using TEM (JEOL Ltd., Japan, JEM-2100F) equipped with an energy dispersive X-ray spectroscopy (EDS) system. The crystal structures of the nanoparticles were analyzed using SR-XRD at the NIMS beamline BL15XU of SPring-8 [24]. The wavelength of the incident X-ray beam for the SR-XRD measurement was 0.06525 nm, which is close to the Nb K absorption edge.

The activation energy barriers of related elementary reactions during methanol decomposition were calculated with the nudged elastic band (NEB) method [25,26] using the PHASE program package (https://azuma.nims.go.jp) to perform first-principle electronic calculations based on DFT. The generalized gradient approximation (GGA) for the exchange-correlation energy was adopted in the calculations [27,28]. A slab consisting of 48 atoms in three atomic layers (each layer = 4 × 4 atoms) was used for the calculations. A vacuum layer of 10 Å was employed for the separation of the slab. The Ni and Sn atoms were fixed in their positions of crystal structures, while the adsorbed phases were movable for structural relaxation. Brillouin-zone integration was performed using the Monkhorst-Pack method [29] with 4 × 4 × 1 k-point sampling mesh. The cut-off energies for the wave function, and the charge density were 350 eV and 1400 eV, respectively. A convergence criterion of 1 × 10^−5^ eV for the total energy and a force criterion of 0.03 eV/Å for the force acting on the atoms were adopted for structural optimization and transition state determination.

## 3. Results and discussion

### 3.1 Characterization of synthesized Ni_3_Sn nanoparticles

The BET specific surface areas of the synthesized Ni_3_Sn and Ni nanoparticles were measured as 9.5 and 4.9 m^2^/g, respectively, which were greater than that of the bulk Ni_3_Sn samples (0.07 m^2^/g) [17]. The average particle sizes of the Ni_3_Sn and Ni nanoparticles were estimated to be 76.7 nm and 137.6 nm, respectively, based on the measured values of the BET specific surface area by assuming a uniform spherical shape of the particles. The BET specific surface areas of the Ni_3_Sn and Ni nanoparticles after hydrogen pre-reduction at 773 K for 1 h were measured to be 4.8 and 1.2 m^2^/g, respectively. The specific surface areas of both samples decreased after pre-reduction, indicating that some agglomeration occurred during pre-reduction.

**Figure 1.**
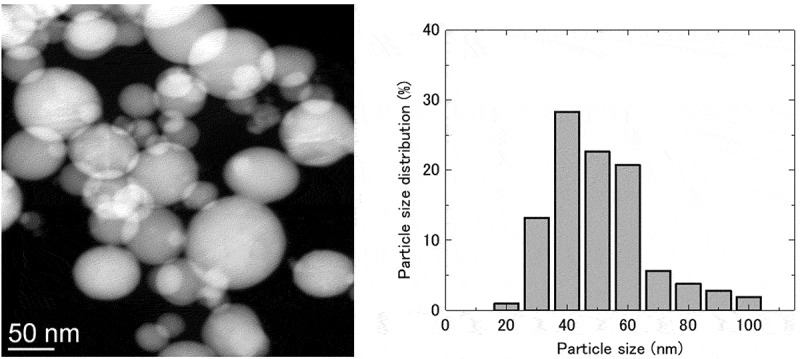
(a) HAADF−STEM image of the synthesized Ni_3_Sn nanoparticles; (b) Distribution of particle sizes obtained by measuring a total of ∼150 particles.

Figure 1(a) shows a high-angle annular dark-field (HAADF) scanning transmission electron microscopy (STEM) image of the Ni_3_Sn nanoparticles. Most particles were observed to be spherical with no obvious porous structure on the surface. A uniform contrast in their brightness suggests a relatively homogeneous chemical composition of the particles because the contrast in HAADF images is highly sensitive to the atomic number of atoms in the sample [30]. Furthermore, the EDS analysis shows that the atomic ratio of Ni and Sn (Ni/Sn) ranged from 2.5 to 3.5 for most particles. Figure 1(b) shows the distribution of particle sizes obtained by observing a total of approximately 150 particles in the STEM images. The distribution of the particle size primarily ranged between 30 to 80 nm, which was generally consistent with the average particle size value of 76.7 nm obtained from the BET surface area measurements.

**Figure 2.**
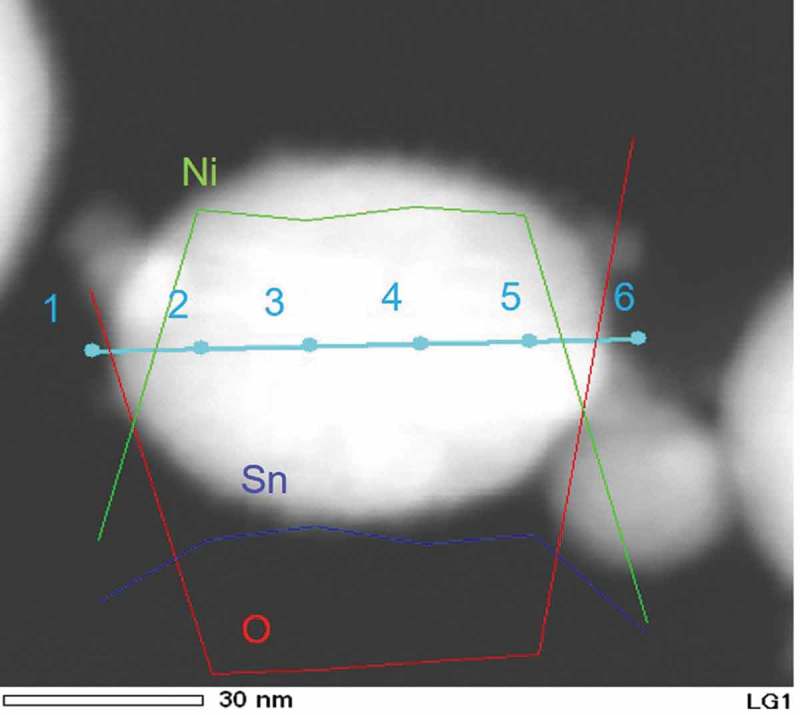
HAADF-STEM image of a single Ni_3_Sn particle, in which EDS line analysis profiles of the Ni, Sn, and O were depicted.

The composition distribution across one particle was estimated by an EDS line analysis. Figure 2 shows the HAADF-STEM image of a single particle, in which EDS line analysis profiles of Ni, Sn, and O are depicted. The quantitative analytical results of the marked positions along the line segment in Figure 2 are summarized in Table 1. The atomic ratio of Ni and Sn (Ni/Sn) across the particle was in a range of 3.0–3.5, which is close to the stoichiometric ratio of Ni_3_Sn, suggesting that the chemical composition in the nanoparticle was highly homogenous. Low concentrations of O (<3.5 at%) within the particle (position Nos. 2–5) suggested minimal oxidation of Ni and Sn occurred in the thermal plasma process.

**Table 1.** EDS analysis results of the synthesized Ni_3_Sn nanoparticles. The positions for the EDS analysis were marked in Figure 2.

Position No.	O (at%)	Ni (at%)	Sn (at%)	Ni/Sn
1 (outside)	63.3	23.3	13.4	1.7
2	1.5	75.8	22.7	3.3
3	1.7	73.8	24.5	3.0
4	2.8	75.7	21.5	3.5
5	3.5	73.9	22.6	3.3
6 (outside)	85.9	7.9	6.2	1.3

**Figure 3.**
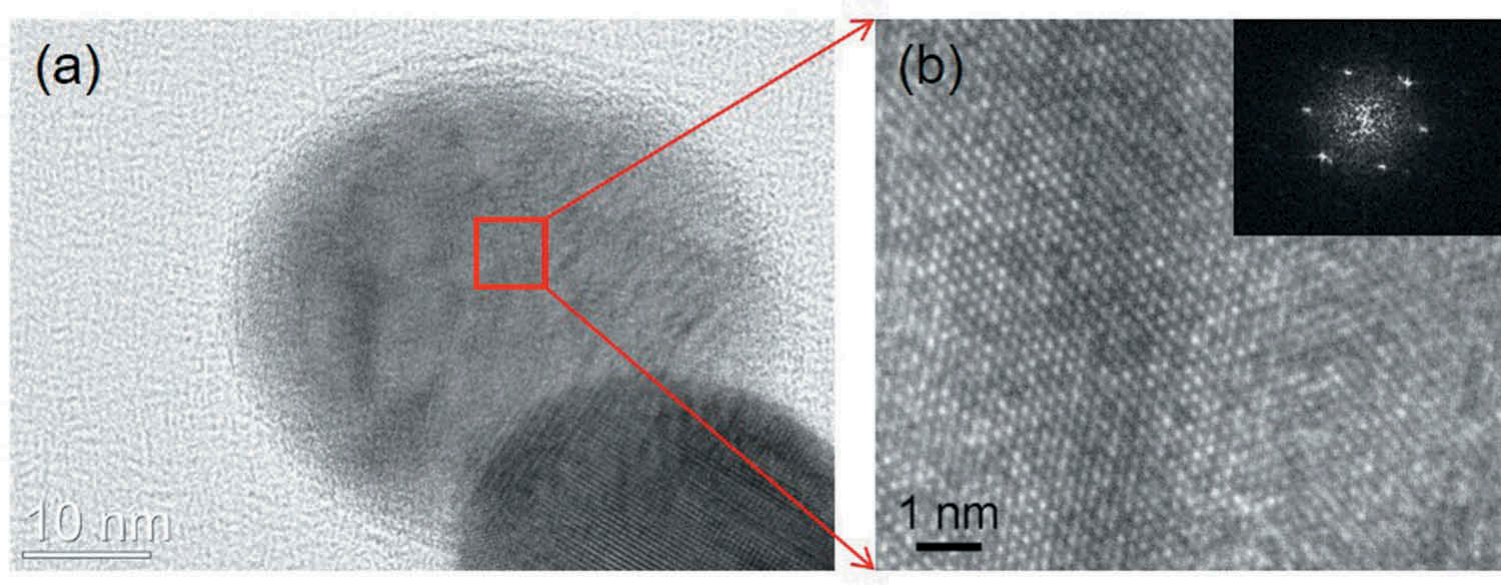
(a) High-resolution TEM image of the Ni_3_Sn particles; (b) Enlarged view of the marked region in (a) with inserting the corresponding fast Fourier transform (FFT) power spectra which were indexed as being [$2\overset{ˉ}{2}1]$ zone axis of Ni_3_Sn phase.

Figure 3(a) shows a high-resolution TEM image of the Ni_3_Sn nanoparticles. Figure 3(b) shows an enlarged view of the marked region in Figure 3(a) with the corresponding fast Fourier transform (FFT) power spectra. The selected diffraction spots were indexed as being [$2\overset{ˉ}{2}1$] zone axis of Ni_3_Sn, confirming the successful synthesis of Ni_3_Sn IMC by the thermal plasma process.

**Figure 4.**
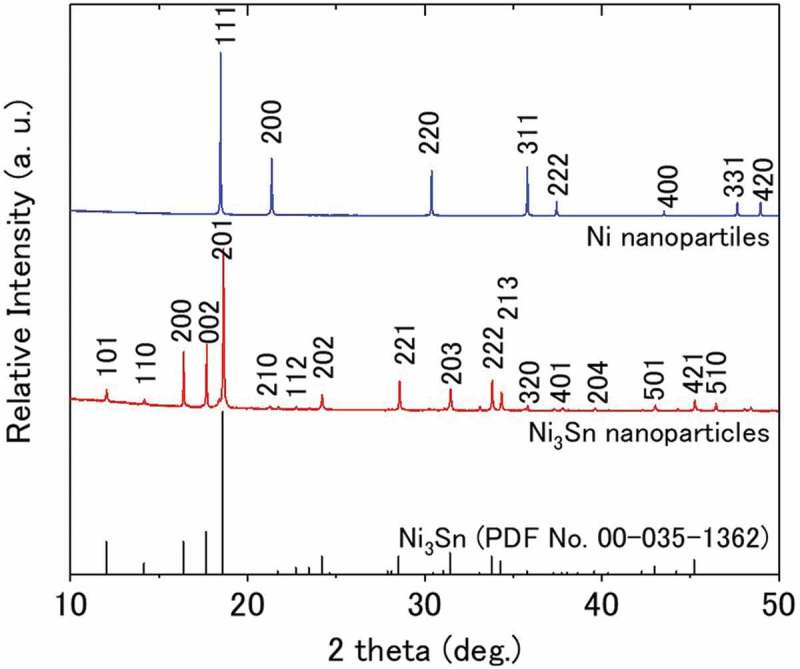
SR-XRD profiles of synthesized Ni_3_Sn and Ni nanoparticles. XRD profile of Ni_3_Sn phase (*P*6_3_ */mmc*, PDF No. 00–035-1362) was inserted for comparison.

The crystal structure of the Ni_3_Sn nanoparticles was further examined by SR-XRD measurement. Figure 4 shows the XRD profiles of Ni and Ni_3_Sn nanoparticles, and the Ni_3_Sn phase (*P*6_3_ */mmc*, PDF No. 00–035-1362) for a comparison. As expected, the diffraction patterns of the Ni_3_Sn nanoparticles corresponded well to that of the Ni_3_Sn phase. The XRD profiles were further qualitatively analyzed by the whole powder pattern fitting method (also referred to as the Rietveld method [31]) using a PDXL2 software package (Rigaku Co.). The synthesized Ni_3_Sn nanoparticles were identified to primarily consist of the Ni_3_Sn phase, together with a small amount of the Ni_0.92_Sn_0.08_ phase (*Fm*-3*m*, PDF No. 01–072-2570). The amount of the Ni_0.92_Sn_0.08_ phase was estimated to be 7.3 mass% by a quantitative analysis. The crystallite size of the Ni_3_Sn and Ni_0.92_Sn_0.08_ phases was 68.3 and 24.4 nm, respectively, as determined by the Williamson–Hall method. For the pure Ni nanoparticles, the XRD profile corresponded perfectly to that of the Ni phase (*Fm*-3*m*), and the crystallite size was estimated to be 56.3 nm. The results of the SR-XRD analyses for the Ni_3_Sn and Ni samples are summarized in Table 2. The refined lattice parameters of Ni_3_Sn phase in the synthesized nanoparticles were a = b = 0.528,612(11), c = 0.424,309(9) nm, which were slightly smaller than the reported values of Ni_3_Sn powder annealed at 1073 K for 88 h: a = b = 0.52,961, c = 0.4281 nm (Ni_3_Sn, PDF Card No. 00–035-1362). This is likely owing to the lower concentration of Sn in the Ni_3_Sn nanoparticles as compared to the stoichiometric composition of Ni_3_Sn, which was identified by the fluorescence X-ray and EDS analyses.

**Table 2.** Results of the SR-XRD analyses for the synthesized Ni_3_Sn and Ni nanoparticles.

					Lattice parameters (nm)			Reliability indexes for Rietveld analysis (%)			
Sample	Phase	Content (mass%)	Crystallite size (nm)	Space group	*a*	*b*	*c*	*R_wp_*	*R_p_*	*R_exp_*	*S*
Ni_3_Sn	Ni_3_Sn	92.7	68.3(0.2)	*P*6_3_*/mmc*	0.528,612(11)	= *a*	0.424,309(9)	4.76	2.82	1.65	2.88
	Ni_0.92_Sn_0.08_	7.3	24.4(2.3)	*Fm-*3*m*	0.35,359(5)	= *a*	= *a*				
Ni	Ni	100	56.3(2.6)	*Fm-*3*m*	0.352,138(3)	= *a*	= *a*	3.56	2.24	0.69	5.17

### 3.2 Catalytic properties for methanol decomposition

**Figure 5.**
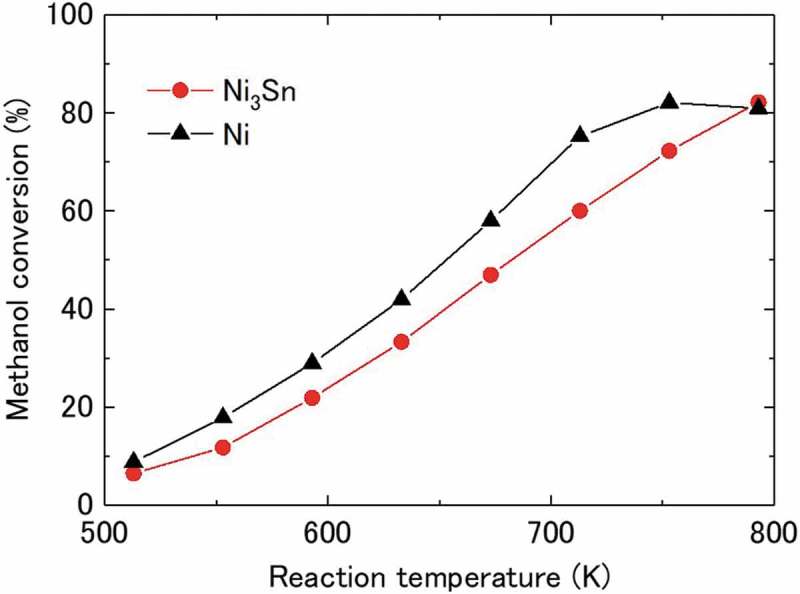
Methanol conversion over Ni_3_Sn and Ni nanoparticles as a function of reaction temperature. CH_3_OH(liquid)/N_2_ = 0.05/30 mL/min, LHSV = 30 h^−1^.

Figure 5 shows the methanol conversion using Ni_3_Sn and Ni nanoparticles as a function of reaction temperature. Methanol conversion increases linearly for Ni_3_Sn throughout the temperature range and reached 82.2% at 793 K. In contrast, for pure Ni, the conversion increased with temperature, reached 82.1% at 753 K, and then slightly decreased to 80.9% with further increasing the temperature up to 793 K. Methanol conversion of Ni_3_Sn was observed to be slightly higher than that of Ni at 793 K; although it was observed to be lower than that of Ni at temperatures below 753 K, suggesting that Ni_3_Sn has a potential advantage applications at high temperatures.

**Figure 6.**
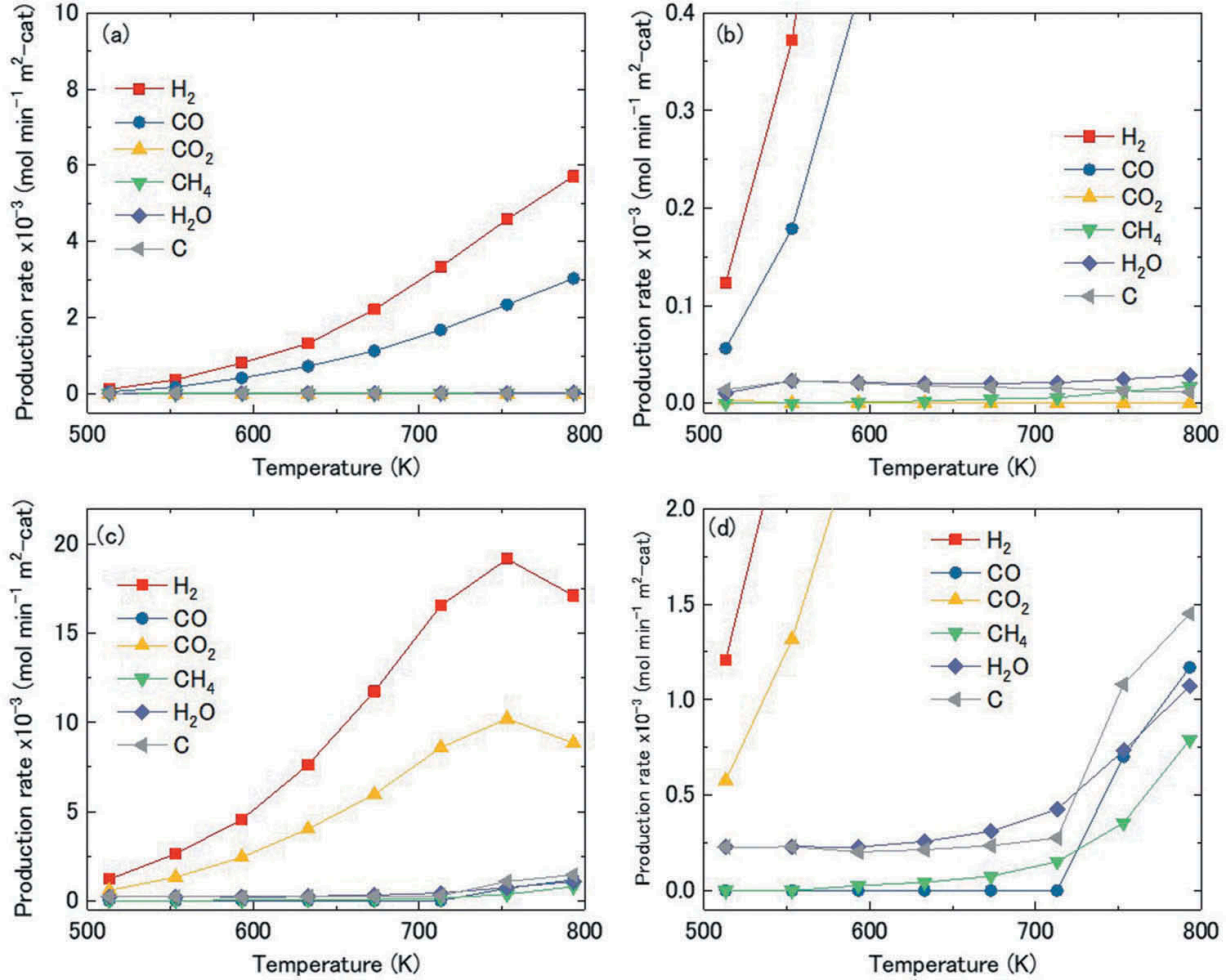
Production rates of outlet products during methanol decomposition over Ni_3_Sn (a) and Ni (c) nanoparticles as a function of reaction temperature; (b) the enlarged view of the region indicated by dotted lines in (a); (d) the enlarged view of the region indicated by dotted lines in (c). CH_3_OH(liquid)/N_2_ = 0.05/30 mL/min, LHSV = 30 h^−1^.

An analysis of the outlet products shows that H_2_ and CO were mainly produced due to the catalytic reaction of both Ni_3_Sn and Ni nanoparticles, accompanied with small amounts of by-products such as CO_2_, C, H_2_O, and CH_4_. The production rates of H_2_, CO, and other by-products were evaluated based on the specific surface areas measured after the hydrogen pre-reduction. Figure 6 shows the production rates of the outlet products as a function of the reaction temperature for Ni_3_Sn (a) and Ni nanoparticles (c). The production rates of H_2_ and CO were significantly higher than those of other products, establishing that the main reaction is methanol decomposition throughout the temperature range Equation (1).

(5) CH3OH← \vboxto.5ex\vss→CO+2H2

The production rates of H_2_ and CO showed a similar temperature dependence to methanol conversion for both Ni_3_Sn and Ni, as shown in Figure 5. Comparison of Figure 6(a,c) indicates that the production rate of H_2_ for Ni_3_Sn is relatively lower than that for Ni, indicating that Ni_3_Sn has a lower catalytic activity for methanol decomposition compared to pure Ni.

The production rates of the by-products of Ni_3_Sn and Ni nanoparticles are more clearly shown in Figure 6(b,d), which are the enlarged views of the regions indicated by dotted lines in Figure 6(a,c), respectively. For Ni nanoparticles, the production rates of H_2_O and C were relatively higher than CO_2_ and CH_4_ at temperatures below 713 K. These production rates rapidly increased with an increase in temperature above 713 K, and the production rates of CO_2_ and CH_4_ simultaneously began to increase significantly. The production rates of all the by-products from Ni_3_Sn nanoparticles were lower than the production rates of by-products from Ni nanoparticles throughout the temperature range, and no obvious increase occurred even at temperatures above 713 K.

The aforementioned by-products were proposed to be generated by the following side reactions.

(6) 2CO→ \vboxto.5ex\vss←CO2+C

(7) CO2+ H2→ \vboxto.5ex\vss←CO+ H2O

(8) CO+3H2→ \vboxto.5ex\vss←CH4 + H2O

Reactions 6, 7, and 8 are commonly referred to as the Boudouard, reverse water-gas shift, and methanation reactions, respectively. The production rate of solid C was estimated by calculating the balance between the reactants and products in the reaction Eqs. 5− 8. The detailed calculation procedure has been described in our previous work [32]. In the case of Ni, the side reactions were assumed to be mainly Boudouard and reverse water-gas shift reactions at temperatures below 713 K; the methanation reaction Equation (5) was assumed to begin in addition to the increasing occurrence of Equations (6) and (7) at temperatures above 713 K, thereby resulting in an increase in production rates of H_2_O, CO_2_, CH_4_, and C. In contrast, in the case of Ni_3_Sn, all the three side-reactions Equations (6)−(8) were suppressed in the whole temperature range, thus resulting in the low production rates of H_2_O, CO_2_, CH_4_, and C.

**Figure 7.**
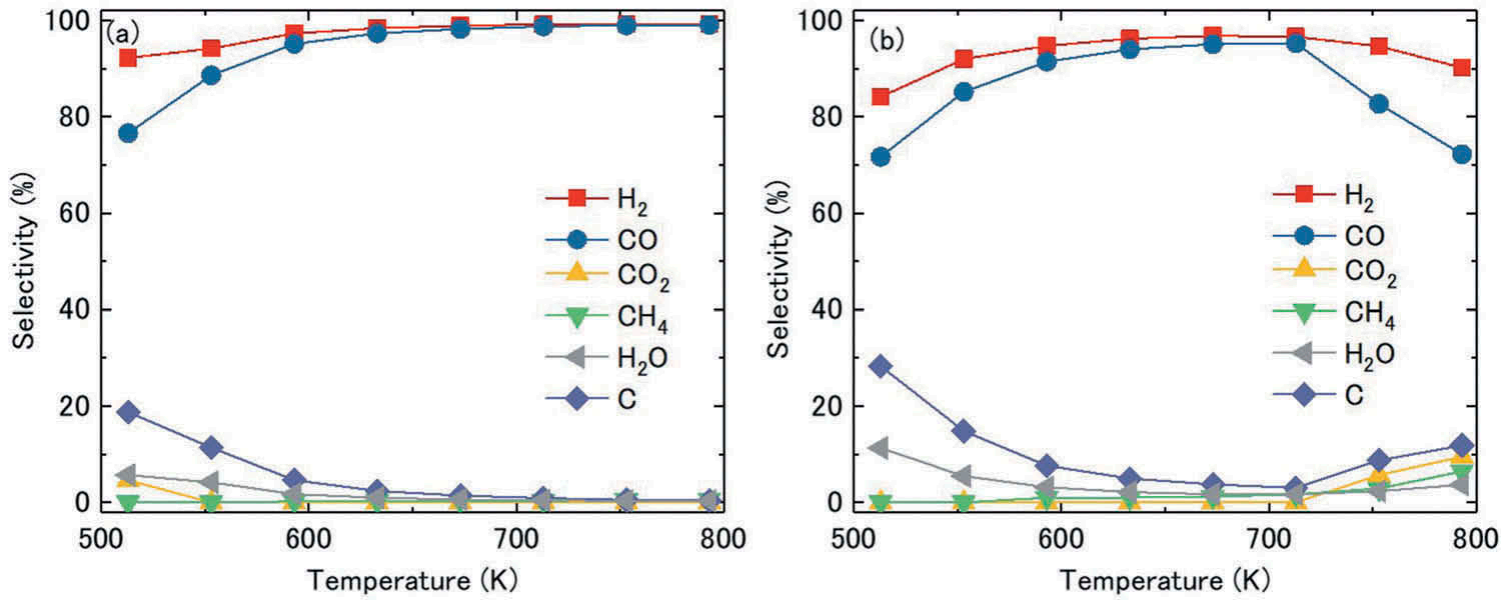
Selectivities toward H_2_, CO, CO_2_, CH_4_, H_2_O, and C in methanol decomposition over Ni_3_Sn (a) and Ni (b) nanoparticles as a function of reaction temperature. CH_3_OH(liquid)/N_2_ = 0.05/30 mL/min, LHSV = 30 h^−1^.

Figure 7 shows the selectivities toward various products as a function of the reaction temperature for Ni_3_Sn (a) and Ni (b). For Ni_3_Sn, the selectivities toward C and H_2_O were ~20% and ~5% at 513 K, respectively, and further decreased to almost zero with the increase in temperature above 673 K. On the other hand, the selectivities toward H_2_ and CO were ~92% and ~78% at 513 K, respectively, increasing linearly and approaching 100% above 673 K. In addition, the selectivity toward CO_2_ decreased rapidly with increasing temperatures, from ~5% at 513 K to almost zero above 553 K. Similarly, the selectivity toward CH_4_ was low in this temperature range. The selectivities toward products follows a similar temperature dependence trend for both Ni and Ni_3_Sn nanoparticles at low temperatures; however the respective values of selectivities differed significantly. That is, the selectivities of pure Ni particles toward C and H_2_O were higher, and the selectivities toward CO and H_2_ were lower compared to the corresponding values of Ni_3_Sn at temperatures below 713 K. At temperatures above 713 K, the selectivities toward H_2_ and CO declined, and the selectivities toward C, H_2_O, CO_2_, and CH_4_ increased, reflecting the increase in the rate of side reactions Equations (6)−(8). These results demonstrate that Ni_3_Sn nanoparticles exhibited a higher selectivity to methanol decomposition than pure Ni nanoparticles. Consequently, the Ni_3_Sn nanoparticles exhibited both high activity and selectivity to methanol decomposition in a wide temperature range, thereby enhancing carbon tolerance and avoiding the consumption of hydrogen by the side-reactions, leading to an efficient hydrogen production.

**Figure 8.**
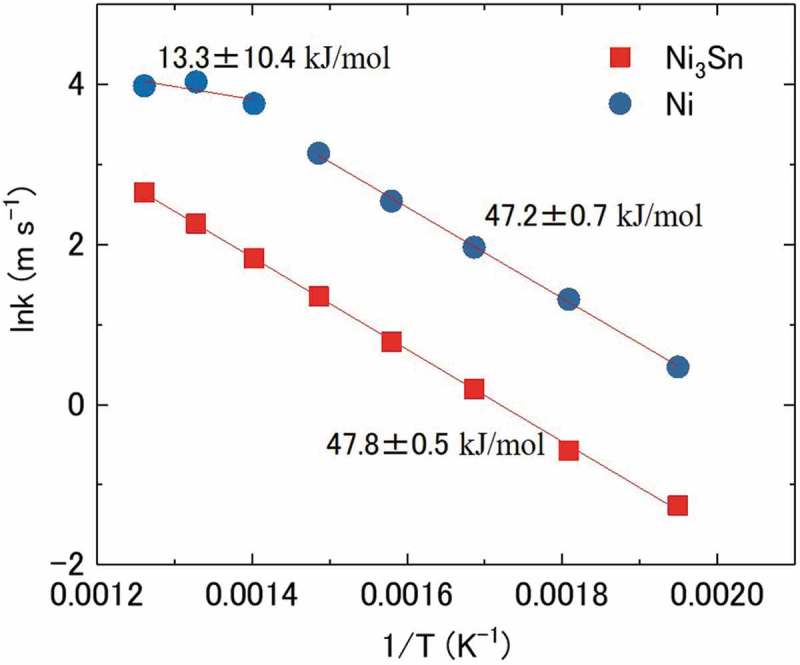
Arrhenius plots of rate constant k and reaction temperature for methanol decomposition over the Ni_3_Sn and Ni nanoparticle catalysts.

The apparent activation energy for methanol decomposition was estimated based on the experimental data at various temperatures for both Ni_3_Sn and Ni nanoparticles by assuming methanol decomposition as a first-order reaction of methanol. Figure 8 shows the Arrhenius plots of rate constant k and reaction temperature for methanol decomposition over Ni_3_Sn and Ni nanoparticle catalysts. A linear approximation was obtained in the investigated temperature range (513 − 793 K) for Ni_3_Sn, and the apparent activation energy for methanol decomposition was estimated to be 47.8 ± 0.5 kJ/mol. For the same experiment and analysis for Ni nanoparticles, a bend was observed at ∼713 K. A linear trend was observed in the low temperature range (513 − 673 K), and the apparent activation energy was estimated to be 47.2 ± 0.7 kJ/mol, which was slightly lower but close to that for Ni_3_Sn nanoparticles. The results suggest that the reaction path of Ni at temperatures below 713 K was similar to that of Ni_3_Sn. On the other hand, the apparent activation energy for Ni lowered to 13 ± 10 kJ/mol at high temperatures (713 − 793 K). The lowering of the apparent activation energy was believed to be related to the increase in the rate of side reactions Equations (6)−(8) at high temperatures (Figures 6 and 7).

**Figure 9.**
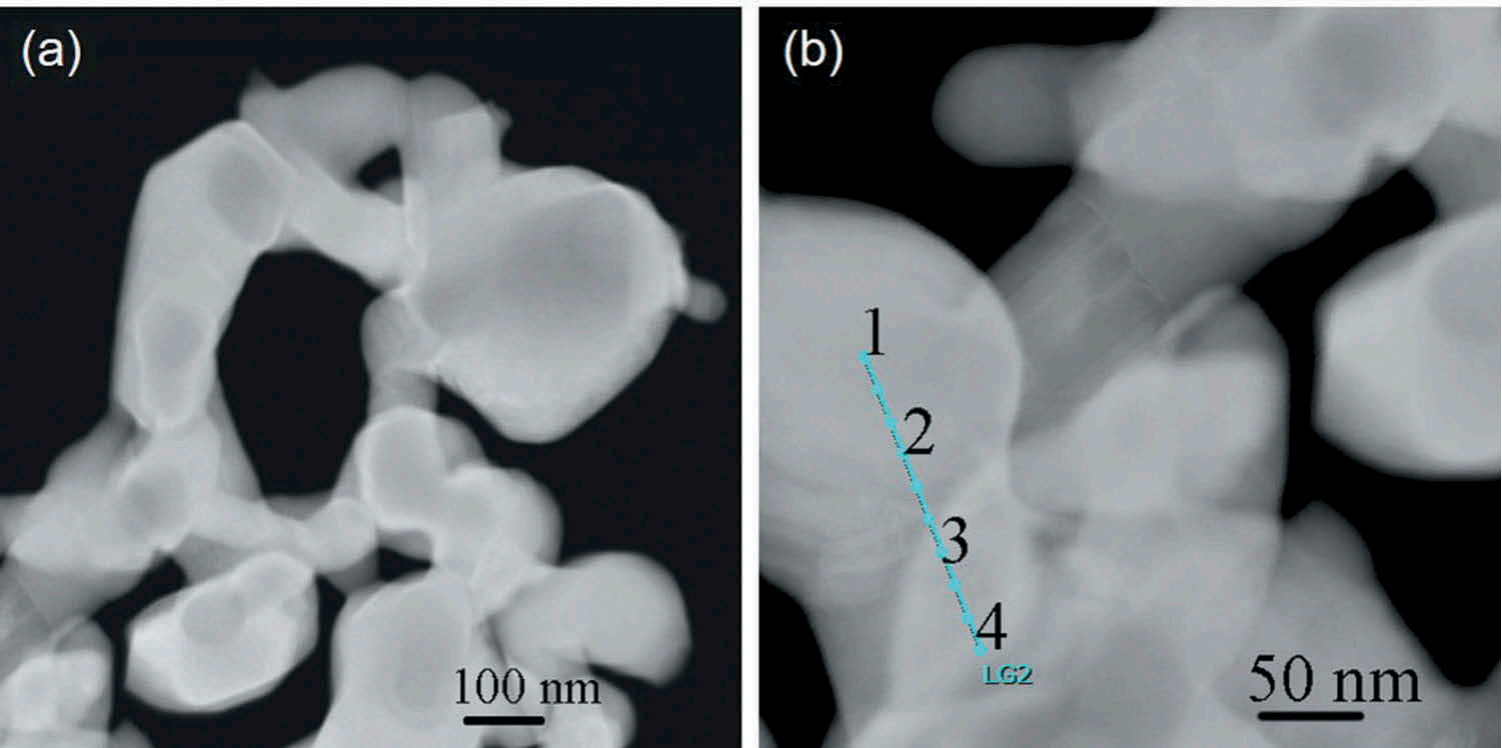
(a) HAADF−STEM image of the Ni_3_Sn nanoparticles after methanol decomposition reaction; (b) the HAADF-STEM image at higher magnification, in which EDS line analysis positions were marked.

The surface morphology and composition of the Ni_3_Sn nanoparticles were examined after the reaction. Figure 9(a) shows the HAADF-STEM image of the Ni_3_Sn nanoparticles after the methanol decomposition test. Figure 9(b) shows the HAADF-STEM image at higher magnification, in which EDS line analysis positions are marked. Some agglomeration of the Ni_3_Sn nanoparticles can be confirmed after the reaction. This agglomeration is possibly attributed to the pre-reduction at 773 K and the subsequent reaction. Table 3 shows the EDS analysis results for the positions marked in Figure 9(b) for the Ni_3_Sn nanoparticles after the reaction. The atomic ratios of Ni and Sn (Ni/Sn) are in the range of 3.1–3.3, and the concentration of O is less than 3.4%. These values are identical to those before the reaction (Table 1), suggesting that the Ni_3_Sn phase has remained unchanged during the reaction. In addition, the concentrations of C are much lower (< 6.6 at%) after the reaction, indicating low carbon deposition over the Ni_3_Sn nanoparticle catalyst.

**Table 3.** EDS analysis results of the Ni_3_Sn nanoparticles after methanol decomposition reaction. The positions for the EDS analysis were marked in Figure 9(b).

Position No.	C (at%)	O (at%)	Ni (at%)	Sn (at%)	Ni/Sn
1	3.4	2.9	71.1	22.5	3.2
2	5.6	2.6	69.5	22.4	3.1
3	6.6	3.0	68.5	21.8	3.1
4	4.6	3.4	69.9	21.2	3.3

### 3.3 DFT calculations for activation energy barriers of CO dissociation and OH formation

As shown above, the Ni_3_Sn nanoparticles exhibit higher selectivity towards H_2_ and CO, and lower selectivity towards C, H_2_O, CO_2_, and CH_4_ as compared to the Ni nanoparticles. To interpret these results at atomic level, we estimate the activation energy barriers of related elementary reactions on the surfaces of their close-packed planes, i.e. Ni_3_Sn (001) and Ni (111). We focus on the following elementary reactions which are inevitable for the formation of C and H_2_O through the side reactions Equations (6)−(8), i.e. CO dissociation Equation (8) and OH formation Equation (9).

(9) $$CO\left(ad\right)\to C\left(ad\right)\;+O\left(ad\right)$$

(10) $$O\left(ad\right)\;+H\left(ad\right)\to OH\left(ad\right)$$

where all the reactants and products are the adsorbed phases on the surface. The initial and final states are first determined by performing structural optimization and calculating adsorption energies of all the phases for both Ni_3_Sn (001) and Ni (111). Subsequently, the transition states are located on the potential energy surface. Figure 10(a,b) show calculated energies for CO dissociation and OH formation during transition state searching on Ni_3_Sn (001) and Ni (111), respectively. The potential energy barrier (E_a_) was determined by calculating the total energy difference between transition state and initial states, and the enthalpy of reaction (∆H) was determined by calculating the total energy difference between final and initial states. The calculated E_a_ and ∆H for these two elementary reactions on Ni_3_Sn (001) and Ni (111) surfaces are summarized in Table 4. The E_a_ for CO dissociation on Ni_3_Sn (001) is estimated to be 396 kJ/mol, while the E_a_ for CO dissociation on Ni (111) is estimated to be 266 kJ/mol which is consistent with the reported E_a_ for CO dissociation on Ni (111) (272 kJ/mol) [33]. The results show that the activation energy barrier for CO dissociation on Ni_3_Sn (001) is higher than that on Ni (111), indicating that after CO is produced by methanol decomposition, it is more difficult for CO to subsequently dissociate to C and O on the surface of Ni_3_Sn than on the surface of Ni. This results in a decrease in carbon deposition and a higher selectivity of CO for Ni_3_Sn compared to Ni (Figure 7).

**Table 4.** Activation energy barriers and enthalpies of reaction for CO dissociation and OH formation on Ni_3_Sn (001) and Ni (111) surfaces.

	Activation energy barrier (kJ/mol)		Enthalpy of reaction (kJ/mol)	
	Ni_3_Sn	Ni	Ni_3_Sn	Ni
CO = C + O	396	266	186	123
O + H = OH	229	132	36	51

E_a_ for OH formation on Ni_3_Sn (001) is 229 kJ/mol, while that for formation on Ni (111) is 132 kJ/mol, as shown in Table 4, indicating that the energy barrier for OH formation on Ni_3_Sn (001) is higher than for that on Ni (111). This implies that it is more difficult to form OH by Equation (10) on the surface of Ni_3_Sn than on the surface of Ni. The formation of OH by direct CH_3_OH dissociation (CH_3_OH → CH_3_ + OH) or C-OH scission on Ni (111) was reported to be difficult during methanol decomposition [33]. Hence, the formation of OH by bonding between O and H Equation (10) is necessary for the formation of H_2_O. Therefore, the formation of H_2_O must be more difficult on Ni_3_Sn than on Ni because of the higher energy barrier for OH formation, resulting in a lower H_2_O selectivity of Ni_3_Sn (Figure 7).

In addition, as shown in Table 4, the enthalpy of CO dissociation on Ni_3_Sn (001) is higher than on Ni (111). CO dissociation is an endothermic reaction, which means that more heat of reaction is required for CO dissociation on Ni_3_Sn (001) than on Ni (111). On the other hand, the enthalpy of OH formation on Ni_3_Sn (001) is lower than on Ni (111), although OH formation is also an endothermic reaction, implying less heat of reaction for OH formation on Ni_3_Sn (001) than on Ni (111). However, the respective enthalpies of OH formation are significantly smaller than the values of the activation energy barrier for OH formation, suggesting that the enthalpy of the reaction is not the main reason for difference in OH formation on Ni_3_Sn (001) and Ni (111). DFT calculations support the experimental results, and give insight into the observation that Ni_3_Sn catalyst suppresses formation of carbon and H_2_O as compared to Ni catalyst.

## 4. Conclusions

Ni_3_Sn nanoparticles were synthesized using a thermal plasma process. The methanol decomposition catalytic properties of the Ni_3_Sn nanoparticles were investigated in a wide temperature range of 513 to 793 K, and also compared with the catalytic properties of pure Ni nanoparticles. Although the Ni_3_Sn nanoparticles showed relatively lower activity than pure Ni nanoparticles, they exhibited a higher selectivity toward H_2_ and CO, and produced less H_2_O, C, CO_2_, and CH_4_ than Ni nanoparticles throughout the investigated temperature range, especially at higher temperatures. DFT calculations revealed that the activation energy barriers for CO dissociation and OH formation were much higher on Ni_3_Sn (001) than on Ni (111), resulting in the high selectivity of Ni_3_Sn toward H_2_ and CO.
